# Role of Coagulation Profile in Predicting Disease Severity Among Patients of COVID-19

**DOI:** 10.7759/cureus.19124

**Published:** 2021-10-29

**Authors:** Animesh Saurabh, Biswajit Dey, Vandana Raphael, Prakash Deb, Yookarin Khonglah, Iadarilang Tiewsoh

**Affiliations:** 1 Pathology, North Eastern Indira Gandhi Regional Institute of Health and Medical Sciences (NEIGRIHMS), Shillong, IND; 2 Anesthesiology and Critical Care, North Eastern Indira Gandhi Regional Institute of Health and Medical Sciences (NEIGRIHMS), Shillong, IND; 3 Internal Medicine, North Eastern Indira Gandhi Regional Institute of Health and Medical Sciences (NEIGRIHMS), Shillong, IND

**Keywords:** activated partial thromboplastin time, prothrombin time, fibrin degradation products, blood coagulation tests, covid-19

## Abstract

Background

Coronavirus disease 19 (COVID-19), caused by severe acute respiratory syndrome coronavirus-2 (SARS-CoV-2), affects the coagulation cascade. In this retrospective study, we aimed to analyze the association of different coagulation parameters including that of D-dimer, fibrinogen, prothrombin time (PT), and activated partial thromboplastin time (aPTT) with severity in COVID-19 patients.

Methodology

A total of 90 patients positive for SARS-CoV-2 on real-time reverse transcription-polymerase chain reaction (rRT-PCR) were included in the study. The patients were categorized as severe and non-severe, and their D-dimer, fibrinogen, PT, and aPTT values on admission were evaluated. The association of the coagulation parameters with disease severity was analyzed by independent t-test and Chi-square test. The cut-off values of these parameters were calculated to predict the disease severity by receiver operator characteristic (ROC) curve.

Results

Out of 90 patients admitted, 42 patients were categorized as severe and the rest 48 patients were categorized as non-severe. D-dimer, fibrinogen, and PT in the severe group were significantly higher than the non-severe group with p-values of <0.001, 0.005, and <0.001, respectively. Cut-off values of 0.99 mg/L for D-dimer,349.5 mg/dL for fibrinogen, and 13.05 seconds for PT were predictive of disease severity among COVID-19 patients.

Conclusion

Severe COVID-19 patients showed significantly higher levels of D-dimer and fibrinogen and prolongation of PT as compared to non-severe COVID-19 patients. Higher levels of D-dimer and fibrinogen, and prolonged PT are predictive of increased disease severity among COVID-19 patients.

## Introduction

Coronavirus disease 19 (COVID-19) is caused by severe acute respiratory syndrome coronavirus-2 (SARS-CoV-2) and primarily affects the respiratory system [1]. COVID-19 is now increasingly regarded as a multisystem disease and affects the hematopoietic and immune systems also. Hematological disorders with a high frequency of thrombotic events are a well-known feature in both COVID-19 and post-COVID-19 patients [2,3].

Severe COVID-19 pneumonia leads to abnormal coagulation profile especially an increase in D-dimer and fibrin degrading products (FDP) [4]. SARS-Cov-2 leads to activation of immune response which causes clearance of the virus [5]. However, in this process of an excessive immune response, an increased amount of inflammatory mediators is released in the body that damage microcirculation causing activation of blood coagulation cascades leading to derangement of coagulation profile [4,6].

Alterations of coagulation parameters during the disease course are intimately associated with COVID-19 severity and mortality. The role of D-dimer in COVID-19 patients has been studied and has been touted as a marker of severity in COVID-19 patients [1,4]. Similarly, fibrinogen has also been studied as a biomarker for disease severity in COVID-19 patients [7]. Prothrombin time (PT) and activated partial thromboplastin time (aPTT) are other coagulation markers that showed alterations in COVID-19 patients especially in severe cases [1].

With this background, we undertook a study to analyze the role of different coagulation parameters including that of D-dimer, fibrinogen, PT, and aPTT in COVID-19 patients.

## Materials and methods

This is a retrospective study done over a period of two months from 1st April 2021 to 31st May 2021. All patients, who were ≥ 21 years of age with COVID-19 confirmed on SARS-Cov-2 real-time reverse transcription-polymerase chain reaction (rRT-PCR) and had routine coagulation profile including d-dimer and fibrinogen done on admission, were included in the study. Patients who were on anticoagulants and patients who had a history of coagulation disorders were excluded from the study.

Blood samples were collected in vacutainers containing 3.2% sodium citrate for coagulation studies. The following coagulation parameters were assessed: PT, aPTT, D-dimer, and fibrinogen. Coagulation profiles including D-dimer and fibrinogen were done on Sysmex CS-2400/2500 automated coagulation analyzer.

The patients were classified into severe and non-severe groups based on symptoms of breathlessness, respiratory rate (RR), and oxygen saturation(SPO2). The patients who were having RR>30/minutes, breathlessness, or SPO2< 90% on room air were categorized as severe and were provided intensive care unit (ICU). Continuous variables were expressed as appropriate means and standard deviation. Categorical variables were summarized as counts and percentages in each category. We grouped the patients into severe and non-severe groups based on the criteria mentioned above. Association between coagulation profile along with D-dimer and fibrinogen was evaluated by independent t-test and chi-square (χ²) test. The receiver operator characteristic (ROC) curve was used to determine blood coagulation profiles including D-dimer and fibrinogen as predictors of severity and a cut-off value was calculated by determining the area under the curve (AUC). A p-value < 0.05 was recognized as statistically significant. All the statistical calculations were performed by statistical package for IBM social sciences (SPSS) software 64-bit version (IBM Corp., Armonk, NY).

## Results

Demographic profile

A total of 90 patients who were positive for SARS-Cov-2 on rRT-PCR were included in the study. The age of the patients ranged from 21 years to 96 years with a mean age of 51.3±16.8 years. There were 53 male and 37 female patients with M:F ratio of 1.43. Out of 90 patients admitted, 42 patients had severe symptoms and required ICU care whereas 48 patients had mild symptoms. The mean age of the patients in the severe group was 57.36±15.3 years as compared to the non-severe group 46±16.4 years with statistically significant results (p-value < 0.001). Co-morbidities were present in 44 (48.89%) out of 90 patients, including diabetes, hypertension, cardiovascular and respiratory disease, cancer and chronic liver, and kidney disease. There was a significant difference between severe and non-severe patients with regard to the presence of co-morbidities (p<0.001) (Table 1).

**Table 1:** Comparison of baseline clinical and coagulation parameters among severe and non-severe COVID-19 patients. PT: prothrombin time; aPTT: activated partial thromboplastin time; SD: standard deviation.

Parameters	Severe (n=42)	Non-severe (n=48)	p-value
Age (mean± SD)	57.36±15.3	46±16.41	0.001
Sex(males/females)	26/16	27/21	0.37
Co-morbidities (yes/no)	29/13	15/33	<0.001
D-dimer in mg/L (mean± SD)	3.1±2.02	0.57±0.52	<0.001
Fibrinogen in mg/dL (mean± SD)	399±134.5	310±157.14	0.005
PT in seconds (mean± SD)	14.6±2.9	12.8±1.4	<0.001
aPTT in seconds (mean± SD)	34±11.3	29±6.004	0.06

Coagulation profile

The mean values of D-dimer, fibrinogen, PT, and aPTT in the severe group were (3.1+2.02) mg/L, (399.5±134.5) mg/dL,(14.6±2.9) seconds, and (34±11.3) seconds, respectively and in the non-severe group were (0.57±0.52) mg/L,(310±157.14) mg/dL, (12.8±1.4) seconds, and (29±6.004) seconds, respectively (Table 1). The difference was statistically significant for D-dimer (p<0.001), fibrinogen (p<0.005), and PT (p<0.001). The difference was not statistically significant for aPTT (p=0.06) (Table 1).

Predictors of severity

**Figure 1:**
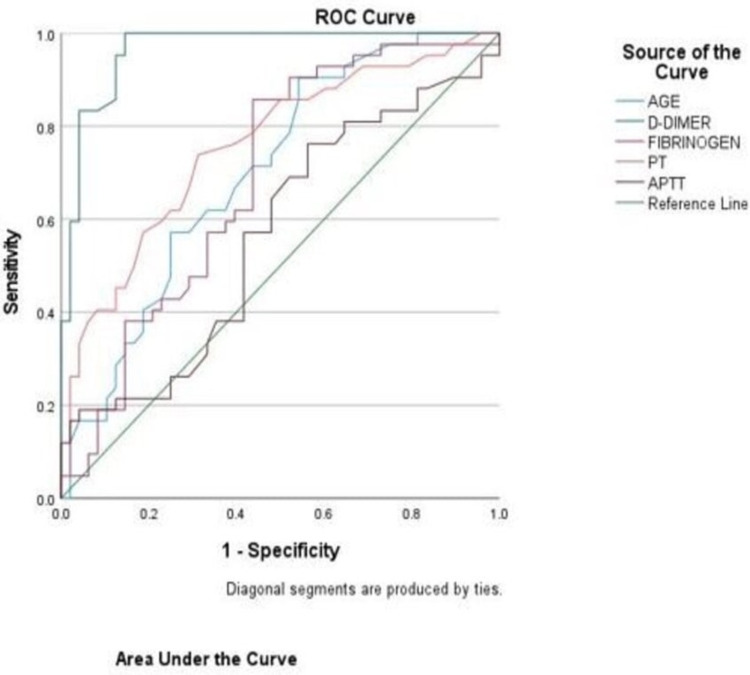
Receiver operating curve (ROC) for ability of age, D-dimer, fibrinogen, PT and APTT to predict severity in COVID-19. PT: prothrombin time; aPTT: activated partial thromboplastin time.

The receiver operating characteristics (ROC) curve was used to determine predictors of severity in COVID-19 by calculating the area under the curve (AUC), sensitivity, specificity, confidence interval, and p-value (Figure 1). A cut-off value was determined by analyzing these data and elaborated in Table 2.

**Table 2:** Area under the curve (AUC) of baseline clinical and coagulation parameters for predicting the disease severity. PT: prothrombin time; aPTT: activated partial thromboplastin time.

Parameters	AUC	95% confidence interval	p-value	Optimal cut-off value	Sensitivity (%)	Specificity (%)
Age	0.70	0.59-0.80	<0.001	48.5 years	66	61
D-dimer	0.964	0.93-0.99	<0.0001	0.99 mg/L	97	86
Fibrinogen	0.68	0.57-0.79	<0.001	349.5 mg/dL	62	61
PT	0.75	0.65-0.85	<0.001	13.05 seconds	73	69
aPTT	0.56	0.448-0.68	0.28	29.5 seconds	57	59

## Discussion

In COVID-19, an abnormal coagulation profile, including elevated D-dimer and fibrinogen levels, has been linked to disease development. In the present study, we analyzed the association of coagulation profile including D-dimer and fibrinogen with disease severity.

The present study showed that the mean age of patients with severe disease (57.36±15.3 years) was significantly higher (p<0.001) than non-severe (46±16.41 years). This finding is similar to that of Zhou et al., who showed that disease severity was more common in older age group patients [8]. However, there was no significant difference between male and female patients based on severity (p-value=0.37) in the present study. Co-morbidities were present in 44 out of 90 patients. Among the 44 patients, 65.9% of severe COVID-19 patients had co-morbidities and 34.1% of non-severe patients had co-morbidities, which was statistically significant (p<0.001). Soni et al. also showed that patients with co-morbidities had increased disease COVID-19 severity [1].

In the present study, we found that the mean value of D-dimer was significantly higher (p <0.001) in severe patients (3.1±2.02 mg/L) than non-severe patients (0.57±0.52 mg/L). In a study by Yao et al., the level of D-dimer was significantly higher in patients with severe COVID-19 [4]. Similarly, a meta-analysis done by Yu et al. proved that severe patients had significantly higher D-dimer value (0.89±0.34 mg/L) than non-severe patients (0.30±0.12 mg/L) [9].

In thrombotic events, the level of D-dimer, which is fibrin-degradation products, is increased suggesting fibrinolysis [4,5]. Several studies have shown that an increase in D-dimer and fibrinogen levels is associated with an increase in disease severity and mortality [4,7]. Increased D-dimer level leads to activation of coagulation cascades secondary to systemic inflammatory response syndrome (SIRS) that cause the formation of microthrombi inside the blood vessels causing disseminated intravascular coagulation (DIC) [5].

In the present study, we observed that D-dimer ≥ 0.99 mg/L predicted increase in disease severity with sensitivity of 97% and specificity of 86% (AUC 0.96; 95% CI = 0.93-0.99, p<0.001). Yao et al. showed that a D-dimer level of > 2.14 mg/L predicted increased disease severity and mortality with a sensitivity of 88.2% and specificity of 71.3% (AUC 0.85; 95% CI = 0.77-0.92) [4]. Zhou et al. also showed that D-dimer > 1mg/L predicted increased disease severity and mortality in patients with COVID-19 [8].

Fibrinogen is a glycoprotein complex that causes the enzymatic conversion of thrombin to fibrin at the tissue injury, causing clot formation and stopping bleeding [10]. Giannis D et al. showed effects of covid-19 on coagulation in-vitro [11]. They found increased expression of genes for the pro-coagulant factor fibrinogen (FGB and FGG) in the virus-infected mononuclear cells. In the present study, the mean value of fibrinogen was significantly higher in severe patients than non-severe patients (399±134.5 vs 310±157.14 mg/dL, p <0.001). According to Di Micco et al., COVID-19 patients with severe disease had a statistically significant increase in fibrinogen levels compared to those with non-severe cases [7].

In the present study, we noted that fibrinogen level >349.5 mg/dL predicted increase in disease severity with a sensitivity of 62% and specificity of 61% (AUC 0.68; 95% CI = 0.57-0.79). Similar findings were corroborated by Zou et al., who showed that 19.1% of patients with severe disease had fibrinogen levels >700 mg/dL compared to 5% in non-severe patients [12].

PT comes as the second most important investigation after D-dimer in COVID-19 patients for diagnosis and management of coagulopathy as per the International Society of Thrombosis and Hemostasis interim guidance [13]. In the present study, the mean value of PT was significantly higher in severe patients than non-severe patients (14.6±2.9 seconds vs 12.8±1.4 seconds, p <0.001). Although the aPTT value was higher in the severe group (34±11.3 seconds) as compared to the non-severe group (29±6.004 seconds), the difference was not statistically significant (p=0.06). Long et al. also showed that the mean value of PT was significantly higher in severe patients than non-severe patients (13.70 ± 3.38 vs 12.34 ± 1.91, p<0.005) [14]. In our study the areas under the ROC curve of PT and aPTT were 0.75 and 0.56 respectively, suggesting PT to be a better predictor of disease severity. Soni et al. also showed the ROC curve of PT and aPTT as 0.741 and 0.663 respectively, suggesting PT to be a better predictor of disease severity and mortality [1]. Other studies also showed that there was no significant difference in aPTT values between patients with mild and severe patients [15].

Selection bias due to the hospital-based population, small sample size, retrospective study design, and absence of serial assessments of D-dimer, fibrinogen, PT, and aPTT were all limitations of this study.

## Conclusions

Severe COVID-19 patients had significantly higher age and presence of co-morbidities as compared to non-severe COVID-19 patients. These patients also showed significantly higher levels of D-dimer and fibrinogen and prolongation of PT as compared to non-severe COVID-19 patients. Higher levels of D-dimer and fibrinogen, and prolonged PT are predictive of increased disease severity among COVID-19 patients. Thus these coagulation parameters on admission are of paramount importance in triaging COVID-19 patients especially those who require aggressive ICU management. This in turn is crucial so that health infrastructure is not oversaturated especially in resource-constrained countries.
